# 1385. Pathway with single-dose long-acting intravenous antibiotic dalbavancin is a cost-saving alternative to usual inpatient care of acute bacterial skin and skin structure infections (ABSSSI)

**DOI:** 10.1093/ofid/ofac492.1214

**Published:** 2022-12-15

**Authors:** Frank Lovecchio, Alasdair D Henry, Quan V Doan, John L Lock, Rosie D Lyles, Xiaolan Ye

**Affiliations:** Arizona State University, University of AZ and Creighton College of Medicines, PHOENIX, Arizona; Genesis Research, Newcastle Upon Tyne, England, United Kingdom; Genesis Research, Newcastle Upon Tyne, England, United Kingdom; AbbVie Inc, Mettawa, Illinois; AbbVie, Evanston, Illinois; AbbVie Inc., Mettawa, Illinois

## Abstract

**Background:**

Acute bacterial skin and skin structure infections (ABSSSI) are a common type of bacterial infection, with the cost of hospitalization being the main contributing factor to overall treatment costs. Two pragmatic clinical trials (Table 1) demonstrated that a new treatment pathway in which patients are treated with the intravenous (IV) long-acting antibiotic, dalbavancin, reduced hospital admission rates and length of stay (LOS) in hospitalized patients. The objective of this study is to evaluate the cost-effectiveness of a single dose of dalbavancin administered in the emergency department (ED) compared with IV antibiotics for appropriate patients who would otherwise be admitted to hospital and receive usual care such as vancomycin or daptomycin.

**Table 1.**
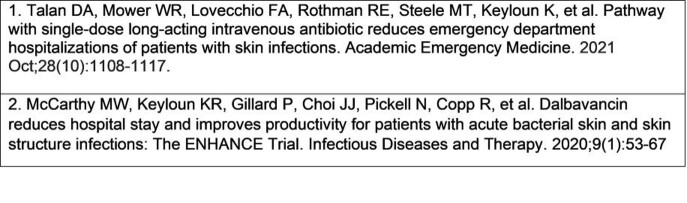
References of the Two Pragmatic Clinical Trials

**Methods:**

A decision-analytic cost-effectiveness model was developed from the perspective of the US healthcare system. The population was ABSSSI patients presenting at the ED, who would be eligible to receive IV antibiotic infusion. Patients can receive IV treatment in the ED, and then discharged or followed by hospital admission for continued management (Fig. 1). A 14-day time horizon was used, representing the typical duration of ABSSSI management. Hospital admission rates and LOS were from the two clinical trials. Cost included ED visits, drug cost, inpatient stay, and physician visits. Input parameter uncertainty was examined via one-way and probabilistic sensitivity analyses.

**Figure 1.**
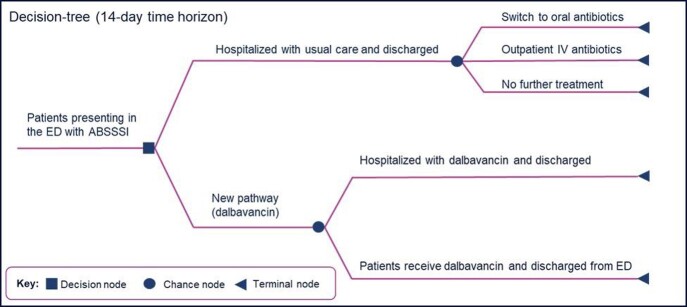
Decision Analytic Model Structure

**Results:**

Providing dalbavancin in the ED resulted in $4,848 savings per patient (2021 USD). The drug cost of dalbavancin treatment was offset by a mean reduction of 4.24 days LOS per patient, which translated as $1,144 savings per hospitalization day avoided. One-way sensitivity analyses demonstrated that the key drivers were the cost of inpatient hospital stay and the LOS with usual care; however, none of the sensitivity analyses resulted in the new pathway being more costly (Fig. 2). Dalbavancin was cost saving in 100% of simulated scenarios.

**Figure 2.**
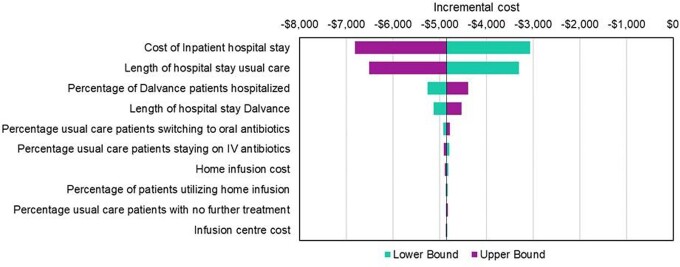
One-way Sensitivity Analysis

**Conclusion:**

These results could help guide the management of ABSSSI by shifting care for appropriate patients from the inpatient to the outpatient setting where patients can be managed successfully, thereby freeing up hospital resources and reducing the total costs of ABSSSI treatment.

**Disclosures:**

**FRANK LOVECCHIO, DO, MPH**, ABBVIE: Speaker|NIH: Grant/Research Support **Alasdair D. Henry, PhD**, AbbVie: Grant/Research Support **Quan V. Doan, PharmD**, AbbVie: Advisor/Consultant|AbbVie: Grant/Research Support|Allergan: Advisor/Consultant|Allergan: Grant/Research Support **John L. Lock, PharmD**, Abbvie: Employee|Abbvie: Stocks/Bonds **Rosie D. Lyles, MD, MHA, MSc**, AbbVie: AbbVie Employee|AbbVie: Stocks/Bonds **Xiaolan Ye, PhD**, AbbVie Inc.: AbbVie Employee|AbbVie Inc.: Stocks/Bonds.

